# Microplastic toxicity: mechanisms, assessment methods, and future research directions

**DOI:** 10.3389/ftox.2026.1766103

**Published:** 2026-02-05

**Authors:** Yifan Zhang, Jiale Ren, Binying Zheng, Jiefang Sun, Jing Zhang, Yumin Niu, Bing Shao, Yushen Jin

**Affiliations:** 1 School of Public Health, Capital Medical University, Beijing, China; 2 Beijing Key Laboratory of Diagnostic and Traceability Technologies for Food Poisoning, Beijing Center for Disease Prevention and Control, Beijing, China; 3 School of Food and Bioengineering, XiHua University, Chengdu, China

**Keywords:** ecological risk assessment, microplastics, omics technologies, oxidative stress, toxicity mechanisms

## Abstract

Microplastics (MPs), plastic particles under 5 mm in diameter, represent a pervasive and persistent global environmental contaminant with cascading adverse effects on aquatic/terrestrial organisms and human health. While existing reviews have summarized isolated aspects of MP toxicity or assessment methods, this review advances the field through three integrated contributions that address critical knowledge gaps. First, it synthesizes physical, chemical, and biological toxicity mechanisms into a unified “particle-environment-organism” cascade, highlighting synergistic interactions that are often overlooked in fragmented syntheses. Second, it provides a critical evaluation of methodological advances by proposing a standardized dosing framework designed to address longstanding challenges to cross-study comparison. Third, it bridges ecological and human toxicology via an integrative conceptual model linking MP properties, environmental modifiers, biological modulating factors, key toxicological events (KTEs) and adverse outcomes, while outlining actionable research priorities and regulatory strategies. By consolidating these elements, this review synthesizes current understanding of MP toxicity and provides a structured framework to enhance comparability across studies, as well as guide future research and regulation. Crucially, it aims to narrow the gap between lab-based findings and real-world application, facilitating the translation of scientific insights into practical strategies for mitigating risks to both ecosystems and human health.

## 1 Introduction

Microplastics (MPs), defined as plastic particles less than 5 mm in diameter, have been recognized as ubiquitous and persistent environmental pollutants (Anic-Vucinic et al., 2025). They originate from diverse sources, including the fragmentation of larger plastic debris (secondary MPs) and the direct release of manufactured microscopic particles used in consumer and industrial applications (primary MPs). The widespread distribution of MPs has been documented across a broad spectrum of ecosystems, from deep marine trenches to remote terrestrial and freshwater environments, highlighting their extensive transport (Kumar et al., 2023; Kushwaha et al., 2024; Morales-Espinoza et al., 2025).

Concerns regarding MP impacts are heightened by their potential for bioaccumulation within organisms and context-dependent biomagnification across trophic levels, posing significant risks to ecosystem integrity and human health (Lombardi et al., 2022; Mercogliano et al., 2020; Rose et al., 2023; Sangkham et al., 2022). MPs can cause direct physical harm and also act as vectors for hazardous substances. This includes the leaching of inherent additives (e.g., plasticizers, flame retardants, stabilizers) and the adsorption of exogenous pollutants (e.g., heavy metals, pesticides, antibiotics), thereby facilitating their entry into biological systems and potentially enhancing toxicological effects under specific environmental conditions (Koelmans et al., 2016; Schiferle et al., 2023; Zhang Y et al., 2025; Zhao YY et al., 2025).

While existing reviews have summarized MP toxicity mechanisms (e.g., Afsa, et al., 2025; Chen et al., 2025; Rafa et al., 2024; Li YF et al., 2025) or assessment methods (e.g., Rosales and Medina, 2025; Liu et al., 2020), most focused on isolated aspects (e.g., aquatic toxicity alone, target organ toxicity or single analytical techniques) and lack a cohesive framework linking particle properties, environmental context, and biological outcomes across scales. Furthermore, few reviews explicitly address the critical gap between lab-based findings and real-world risk assessment—such as the disconnect between high experimental doses and environmentally relevant concentrations, or the challenge of extrapolating ecological data to human health impacts.

**FIGURE 1:**
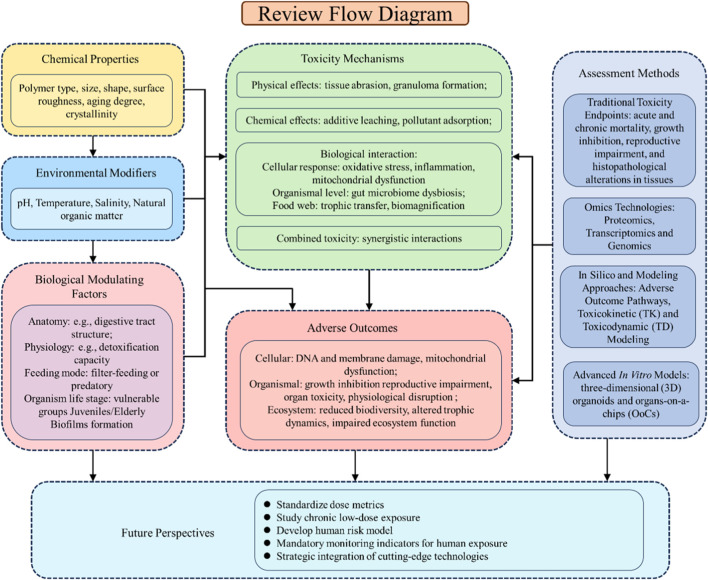
Conceptual framework of microplastic toxicity: from drivers to adverse outcomes and future directions.

This review fills these gaps with three integrated contributions: First, it synthesizes physical, chemical, and biological toxicity mechanisms into a unified “particle-environment-organism” cascade, emphasizing synergistic interactions that are often overlooked in fragmented syntheses. Second, it provides a critical evaluation of methodological advances—from traditional endpoints to omics and computational modeling—by highlighting their complementary strengths and limitations, rather than merely listing techniques. This includes a proposed standardized dosing framework (particle number vs. mass-based metrics) tailored to different research questions, designed to address longstanding challenges to cross-study comparison. Third, it bridges ecological and human toxicology by outlining actionable regulatory strategies and future research priorities that directly inform evidence-based policy, moving beyond descriptive knowledge gaps to solutions-oriented insights. Consequently, this review aims to consolidate recent advances in understanding MP toxicity mechanisms (including physical damage, chemical leaching, vector effects, and the induction of complex biological responses such as oxidative stress and inflammation) and the evolution of toxicity assessment methodologies, while identifying critical knowledge gaps and suggesting future research directions to enhance risk assessment frameworks and inform mitigation strategies for MP pollution (Figure 1).

## 2 Methodology

This review focused on studies of MPs and nanoplastics (NPs, <1 μm) conducted on environmental and laboratory scales to evaluate their toxicological mechanisms, assessment methodologies, and hazardous consequences. Literature was searched in May 2025, using Web of Science (WoS) and Scopus databases. The search was restricted to peer-reviewed original papers and reviews written in English, with no temporal limitations to ensure comprehensive coverage of foundational and recent advances. The search terms were used in WoS and Scopus: (“microplastic” OR “micro plastic” OR “nanoplastic” OR “nano plastic”) AND (“toxicity mechanism” OR “exposure” OR “risk assessment” OR “omics” OR “computational modeling” OR “environmental effect” OR “human health” OR “bioaccumulation”). The information gathered from the literature was then thematically organized into subtopics: toxicity mechanisms (physical, chemical, biological, and combined effects), methodological advances (traditional endpoints, omics technologies, *in silico* modeling, and advanced *in vitro* systems), critical knowledge gaps, and actionable regulatory strategies and future research priorities.

## 3 Mechanisms of microplastic toxicity

The toxicity of MPs is not intrinsically attributed to a single mechanism, but arises from a complex interplay of particle properties, environmental conditions, biological systems, and the synergistic interactions of physical, chemical, and biological effects that collectively modulate toxicological outcomes (Table 1).

**TABLE 1:** MP exposure parameters and toxicological effects.

Polymer type (size)	Experimental model (*in vivo/in vitro*)	Exposure methods & dose	Accumulation site/Target organs	Toxicity response	References
PS: 50 nm, 100 nm, 500 nm (COOH-modified)	Mammal (BALB/c mice, *in vivo*)	Oral exposure; 10 mg/kg and 1 mg/kg, once a day for 28 consecutive days	Intestinal absorption →spleen, liver, and heart (50, 100, 500 nm); testis and kidney (50 nm)	Disrupted intestinal barrier; liver, heart and kidney toxicity	Du et al. (2024)
PS: 42 nm	Mammal (male C57BL/6 J mice, *in vivo*); cell (hCMEC/D3 cells, *in vitro*)	Oral exposure; 0.5–50 mg/kg. for 7 days	Brain	Microglia activation and neuron damage in brain of mice; oxidative stress, inflammatory response, and necroptosis in hCMEC/D3 cells	Shan et al. (2022)
PS: 52 nm, 53 nm, 57 nm, 58 nm, 120 nm, 180 nm, 330 nm (NH_2_-modified, positively charged)	Aquatic vertebrate (Fish, *in vivo*)	Waterborne exposure	Blood-brain barrier penetration	Brain damage; behavioral disorders (reduced locomotor activity)	Mattsson et al. (2017)
PS: 2 μm (carboxy and amine functionalized)	Aquatic invertebrate (*Daphnia magna,* *in vivo*)	Waterborne exposure; 0.26–5.2 mg/L	Gut	Significant metabolic perturbations and increased activities of LDH and GST (carboxy functionalized microparticles showed higher toxicity)	Panagiotidis et al. (2023)
PS: 2 μm and 100 nm (carboxy and amine functionalized)	Aquatic invertebrate (*Daphnia magna,* *in vivo*)	Waterborne exposure; 0.1, 0.5 and 1 mg/L for 21 days	Gut	Decreased egestion and feeding rate (100 nm)	Rist et al. (2017)
PS: i100 nm and 1,300 nm	Terrestrial invertebrate (*Eisenia fetida,* *in vivo*)	Soil exposure; 100 and 1,000 μg/kg soil for 14 days	Intestinal lumen	Intestinal cell damage; oxidative stress; histopathological changes of intestinal tissue; DNA damage	Jiang et al. (2020)
PE: 10–50 μm	Mammal (ICR mice, *in vivo*); cell (human-derived PBMCs and HMC-1 cells, *in vitro*)	Oral exposure; 500, 1,000, and 2000 mg/kg/day for 28 days	Alveolar tissues	Granulomatous inflammation; pro-inflammatory cytokine (TNF-α, IL-6 and IL-1β) release	Lee et al. (2022)
PS: fibre-/fragment-shaped (8.9 ± 10.1 µm by 1.14 ± 0.97 µm) and spherical (200 nm and 2 μm)	*In vitro* (Caco-2 cell monolayer)	Cell culture exposure; 0–100 μg/mL	Cell	Decreased the intracellular H_2_O_2_ levels; redox response; membrane damage	Saenen et al. (2023)
PS: 1–10 µm	Aquatic vertebrate (carp, *in vivo*)	Waterborne exposure; 1,000 ng/L for 21 days	Heart tissues	Myocardial inflammation; cell death via TLR4/NF-κB pathway activation	Zhang et al. (2023)
PS: 100 nm	Aquatic vertebrate (red tilapia, *Oreochromis niloticus,* *in vivo*)	Waterborne exposure; 1–100 μg/L (combined with 50 μg/L roxithromycin, ROX) for 14 days	Gut, gills, brain, and liver	Enhance the bioaccumulation of ROX in various tissues and mitigate the oxidative damage that caused by ROX	Zhang et al. (2019)
PS: 15 μm (beads) and 4–40 μm (fragment) Polypropylene: 20 μm in diameter and 20–100 μm in length (fibers)	Aquatic vertebrate (zebrafish, *in vivo*)	Waterborne exposure; 20 mg/L for 7 days	Intestine (fibers > fragments > beads)	Intestinal toxicity (fibers > fragments > beads): mucosal damage, increased permeability, inflammation, metabolism disruption and microbiota dysbiosis	Qiao et al., 2019
PS: 10 μm-100 nm	Mammal (BALB/c mice, *in vivo*)	Oral exposure; 0.1 mg/day for 7 days	Colon	NPs induce ferroptosis-mediated immunogenic cell death and MPs cause cell metabolic reprogramming	Cheng et al. (2025)
PS: 0.1 μm and 5 μm	Aquatic invertebrate (*Daphnia magna,* *in vivo*)	Waterborne exposure; 0.1 mg/L and 1 mg/L	Intestine (bioaccumulation: 0.1 μm > 5 μm)	Disrupting glucose metabolism and intestinal structure; gut microbiome dysbiosis; alter life history	Zhao BY et al. (2025)
PS: 1 μm	Mammal (C57BL/6N mice, *in vivo*)	Waterborne exposure; 1 mg/L and 5 mg/L for 12 weeks	Liver	Kupffer cell polarization imbalance; hepatic lipid accumulation	Li T et al. (2025)
PS: 0.5 μm	Mammal (BALB/c mice, *in vivo*)	Waterborne exposure; 1 mg/L (combined with 100 mg/L Cd) for 12 weeks	Liver	Cell death; inflammation; fibrosis	Li YH et al. (2025)
PS and PE: 564 μm (PS), 622 μm (high-density PE) and 632 μm (low-density PE)	*In vitro* (human coronary artery smooth muscle cells)	Cell culture exposure; 1 mg/mL for 3 and 7 days	Cell	Pro-inflammatory cytokine (TNF-α, IL-6) release; oxidative stress; inflammatory signaling activation	Lomonaco et al. (2024)
PS: 25 and 50 nm	Aquatic invertebrate (*Caenorhabditis elegans*)	Medium exposure; 100 μg/L for 36 h (25 nm) and 0–1,000 μg/L for 72 h (50 nm)	Whole body	Inhibition in body length, survival rate, head thrashes, and body bending; oxidative stress; cellular damage; neurodevelopmental toxicity; locomotor dysfunction	Shang et al. (2021)

### 3.1 Physical effects and governing particle properties

The intrinsic physical and chemical properties of MPs are primary determinants of their biological interactions, environmental behavior, and potential for physical harm. Polymer type (e.g., polyethylene-PE, polypropylene-PP, polystyrene-PS) determines key characteristics such as buoyancy, surface chemistry, and susceptibility to degradation (Tiago et al., 2025; Zhang XY et al., 2024), while polymer crystallinity influences rigidity and the potential for additive leaching. Aging driven by environmental weathering (e.g., UV exposure, abrasion, microbial action) alters surface properties by increasing roughness, creating cracks, and introducing oxygen-containing functional groups (e.g., carbonyl, hydroxyl) (Tiago et al., 2025; Tumrani et al., 2025), which in turn enhances adsorption capacity for co-contaminants and modulates physical interactions with biological systems (Tumrani et al., 2025).

Size, shape, and surface roughness are critical for direct physical toxicity (Choi et al., 2021), with distinct patterns observed between MPs (1 μm-5 mm) and NPs (<1 μm), and inconsistencies in translocation outcomes attributed to contextual variables (test system, detection method, exposure condition and species-specific biology)—factors that clarify the strength of evidence for each particle class:

NPs consistently demonstrate greater capacity to cross biological barriers (intestinal epithelium, gill lamellae, blood-brain barrier, placental barrier) across many taxa, including mammals (e.g., mouse intestinal absorption and brain accumulation of 50 nm PS NPs; Du et al., 2024; Shan et al., 2022), aquatic vertebrates (e.g., zebrafish larval and fish blood-brain barrier penetration by ≤100 nm polyethylene NPs; Lei et al., 2023; Mattsson et al., 2017), and invertebrates (e.g., *Caenorhabditis elegans* cuticle crossing by 50 nm PS NPs; Shang et al., 2021). This is mechanistically linked to their small size (enabling paracellular or transcellular transport) and high surface area-to-volume ratio (enhancing interactions with membrane receptors). These findings rely on advanced imaging (e.g., fluorescence microscopy, hyperspectral microscopy) (Mattsson et al., 2017; Shang et al., 2021) and quantitative analytical techniques (e.g., inductively coupled plasma-mass spectrometry for metal-labeled NPs) (Novak et al., 2025), which minimize false-negative results by directly tracking NP localization in tissues.

MPs larger than 1 μm rarely cross intact biological barriers in most species; instead, they predominantly accumulate in the gastrointestinal (GI) tract or gill tissues (e.g., 2 μm PS MPs are retained in the guts of *Daphnia magna* without systemic translocation; Panagiotidis et al., 2023; Rist et al., 2017). Exceptions are only observed in species with specialized epithelial structures (e.g., filter-feeding bivalves with permeable gill epithelia) (Mkuye et al., 2022) or when MPs undergo partial degradation into smaller fragments (<1 μm) *in vivo*.

The internalization of MPs causes direct physical damage, with findings stratified by model system (*in vivo* vs. *in vitro*) and taxonomic group to clarify the key adverse outcomes.

#### 3.1.1 GI tissue abrasion

Gastrointestinal (GI) tissue abrasion induced by MPs are supported by robust *in vivo* evidence across diverse taxa, including aquatic invertebrates (e.g., *D.aphnia magna* exposed to irregular PS microspheres; Zhao BY et al., 2025), teleost fish (e.g., intestinal epithelial injury in zebrafish exposed to PS MPs with different shapes: fibers > fragments > beads; Qiao et al., 2019; Cheng et al., 2025), and terrestrial invertebrates (e.g., intestinal cells damage in *Eisenia fetida* exposed to PS MPs; Jiang et al., 2020). In contrast, *in vitro* evidence remains limited: while gut epithelial cell (Caco-2) monolayers exhibit elevated permeability and membrane damage following exposure to micron-sized spherical and fiber-and fragment-shaped PS MPs (Saenen et al., 2023), such cellular-level responses fail to fully recapitulate the tissue-level ulceration observed in living organisms.

#### 3.1.2 Inflammation and granuloma formation

Inflammation and granuloma formation triggered by MPs are underpinned by strong *in vivo* evidence across multiple taxa: MPs (e.g., polyester, polypropylene) induce prolonged tissue retention and robust inflammatory responses in aquatic organisms (e.g., myocardial inflammation in carp; Zhang et al., 2023) and mammals (e.g., granulomatous inflammation in the alveoli of ICR mice after oral exposure to polyethylene MPs; Lee et al., 2022). Complementing these *in vivo* findings is emerging *in vitro* evidence: vascular smooth muscle cells exhibit the release of pro-inflammatory cytokines (TNF-α, IL-6) upon co-culture with aged PS MPs, which provides a cellular-level mechanistic basis for the inflammatory processes observed in living organisms (Lomonaco et al., 2024).

Notably, the threshold for physical damage remains poorly defined across taxa, with few studies quantifying dose-response relationships between particle shape/size and injury severity—a critical gap for risk assessment.

### 3.2 Chemical effects and environmental modulating conditions

MPs exert chemical toxicity through two primary routes: the leaching of inherent additives and the adsorption of exogenous pollutants. Their chemical behavior and toxic potential are significantly influenced by the surrounding environmental matrix (Rafa et al., 2024; Yu et al., 2024). Many plastics contain non-covalently bound additives (e.g., plasticizers, stabilizers, flame retardants, colorants), which can leach out under environmental conditions (Corami et al., 2022; Gulizia et al., 2023; Yu et al., 2024). Concurrently, MPs act as vectors for a wide spectrum of exogenous pollutants, including heavy metals (e.g., lead, cadmium) (Luo et al., 2025; Wang JY et al., 2025), pesticides (e.g., DDT) (Fu et al., 2024; Zeng et al., 2024), and antibiotics (Xia et al., 2025). Adsorption capacity is governed by both MP properties (polymer type, aging degree, crystallinity) and key environmental parameters (He et al., 2025; Huo et al., 2025; Koelmans et al., 2016; Wang et al., 2021).

Critical environmental conditions regulate MP chemical toxicity by modifying MP-pollutant interactions in external matrices, and these effects are further amplified or modified within the gastrointestinal tract microenvironment—a dynamic interface where environmental variables converge with biological factors (e.g., low pH, digestive enzymes, bile salts, surfactants) to govern contaminant desorption and bioavailability. Key environmental modifiers and their link to gut-mediated effects include:

1. pH: External aquatic/soil pH shapes MP surface charge and pollutant ionization (e.g., protonation of heavy metal ions or deprotonation of pesticide functional groups), which directly influences subsequent desorption in the gastrointestinal tract. For example, the strongly acidic environment of the vertebrate stomach (e.g., pH ∼1.5–3.5) can enhance the desorption of cationic pollutants (e.g., lead, cadmium) from microplastics. The mechanism involves the protonation of MP surface functional groups (e.g., carboxyl, hydroxyl), disrupting MP-pollutant electrostatic bonds (Tang et al., 2025; Gao et al., 2023).
2. Temperature: Fluctuations in external temperature accelerate MP aging and increase additive leaching rates in environmental media (Mao et al., 2024). Thus, once ingested, core body temperatures (e.g., 37 °C in mammals, 25 °C–30 °C in aquatic ectotherms) further amplify leaching kinetics of additives (e.g., phthalates, flame retardants) and desorption of adsorbed pollutants by increasing molecular diffusion within the gut lumen.
3. Salinity: In aquatic systems, higher ionic strength promotes MP aggregation (Shams et al., 2020), reducing bioavailability to pelagic organisms but increasing exposure for benthic feeders. Within the gastrointestinal tract, physiological salinity (e.g., 0.9% in mammalian intestines, approaching seawater levels of ∼3.5% in marine fish) modulates MP-pollutant interactions: elevated ion concentrations (e.g., Na^+^, Cl^−^) compete with adsorbed contaminants for binding sites on MP surfaces (Fred-Ahmadu et al., 2020), enhancing desorption while also influencing gut epithelial permeability to released toxicants.
4. Natural organic matter (NOM): In external environments, NOM forms a “bio-corona” on MP surfaces, altering pollutant adsorption affinity (Ali et al., 2024). In the gastrointestinal tract, this NOM corona interacts with bile salts and digestive surfactants (e.g., phospholipids, fatty acids), either stabilizing MP-pollutant complexes (reducing desorption) or disrupting the corona (increasing bioavailability of both additives and adsorbed pollutants) (Kihara et al., 2025).

Upon ingestion, these environmental-biological interactions drive targeted contaminant desorption in the gut, forming elevated local concentrations (Zhou et al., 2020) that enhance absorption across the intestinal epithelium. This process can amplify adverse effects—including endocrine disruption, neurotoxicity, hepatotoxicity, and metabolic disturbances—beyond those from individual contaminant exposures, through the magnitude of amplification varies by organism, contaminant type, and exposure duration (Paul-Pont et al., 2016; Zhang et al., 2019). However, most studies on combined toxicity use artificial exposure concentrations (e.g., 1–100 mg/L for microplastics in aquatic models, equivalent to millions to hundreds of millions of particles per cubic meter) (Zheng et al., 2025; Li et al., 2021) that are 1-3 orders of magnitude higher than environmentally relevant levels (typically ng/L to μg/L or hundreds to tens of thousands of particles per cubic meter in surface waters) (Qu et al., 2023; Zhao SY et al., 2025; Desforges et al., 2014), making it difficult to extrapolate results to environmental risk assessment. Additionally, the relative contribution of additive leaching versus exogenous pollutant adsorption to overall chemical toxicity remains unresolved in many organisms, particularly under chronic low-dose exposure scenarios that mimic real-world conditions. To better bridge this gap, we propose a context-dependent dose metric framework (detailed in Section 5.1): prioritizing particle number-based doses for investigating mechanistic questions related to physical interactions (e.g., membrane translocation, tissue abrasion), and mass-based doses for assessing chemical effects (e.g., additive leaching, pollutant adsorption).

### 3.3 Biological interactions and species-specific susceptibility

Toxicological outcomes at cellular, organismal, and food web levels depend heavily on biological context, with species-specific susceptibility and subcellular cascades driving adverse effects. At the cellular and subcellular level, MPs trigger a cascade of harmful biological responses. A primary mechanism is oxidative stress induction, characterized by excessive reactive oxygen species (ROS) generation (Zhang QR et al., 2025; Zhao BT et al., 2025); overwhelming antioxidant defenses leads to lipid peroxidation (membrane damage), protein oxidation (enzyme dysfunction), and DNA damage (potentially causing mutations and carcinogenesis) (Islam et al., 2025; Jiang et al., 2020). MPs also disrupt essential metabolic pathways, causing mitochondrial dysfunction and impaired energy homeostasis that impacts growth, reproduction, and overall vitality (Fan et al., 2025; Ma et al., 2025; Song et al., 2025), while certain MPs activate pro-inflammatory and stress-related signaling pathways (e.g., MAPK, PI3K-AKT/mTOR, NF-κB) regulating inflammation, apoptosis, and immune responses in aquatic and mammalian models (Li T et al., 2025; Qian QH et al., 2025; Song et al., 2025), potentially leading to chronic inflammation, immunosuppression, or autoimmune reactions.

At the organismal level, susceptibility varies with species-specific anatomy (e.g., digestive tract structure), physiology, and detoxification capabilities (Cui et al., 2025; Sanchez-Guerrero-Hernandez et al., 2023); filter-feeding organisms face higher exposure risk due to their feeding mechanisms (Sanchez-Guerrero-Hernandez et al., 2023). The interactions between MPs and host microbiomes are an emerging critical factor: MP ingestion alters gut microbiota composition, causing dysbiosis that impairs immune function and nutrient absorption, indirectly amplifying toxicity (Huang et al., 2025; Lin et al., 2025). However, studies on microbiome effects are largely descriptive, with few investigating causal links between dysbiosis and adverse organismal outcomes (e.g., reduced growth, reproductive impairment). Conversely, microbial biofilms on MP surfaces can influence MP environmental fate and pollutant degradation (He et al., 2022; Moyal et al., 2023; Ventura et al., 2024), but the extent to which biofilm formation modulates MP toxicity is poorly understood.

At the food web level, trophic transfer—where MPs and associated chemicals move from prey to predator—drives context-dependent-bioaccumulation and biomagnification, exposing higher trophic levels including humans to elevated contaminant concentrations (Gao SK et al., 2024). Yet, evidence for biomagnification is inconsistent across ecosystems: while some marine food webs show clear biomagnification (Mercogliano et al., 2020), terrestrial food webs have yielded mixed results (Kumar et al., 2023). This inconsistency may reflect differences in MP bioavailability across matrices or methodological challenges in quantifying MP transfer between trophic levels.

### 3.4 Combined toxicity

Interactions between MPs, their inherent additives, adsorbed exogenous pollutants, and biological systems often result in synergistic toxicity, where the combined effect exceeds the sum of individual effects. MPs facilitate co-transport of pollutants (e.g., heavy metals, antibiotics) into organisms, altering their bioavailability, distribution, and toxicokinetics (Suljevic et al., 2025; Wang et al., 2021). For example, MPs can increase the uptake and reduce the elimination rate of co-pollutants, thereby potentiating their toxicity (Suljevic et al., 2025). However, current methods for assessing combined toxicity are largely limited to binary mixtures (MP + single pollutant), failing to capture the complexity of real-world exposures where MPs interact with multiple contaminants simultaneously. This limitation undermines the accuracy of ecological risk assessment frameworks, which typically focus on single contaminants rather than complex mixtures (Liu et al., 2025). Integrating particle, environmental, and biological modulating factors to understand these synergistic interactions is crucial for accurate prediction of the environmental impact of MP pollution.

## 4 Methodological advances in toxicity assessment

The assessment of MP toxicity is evolving rapidly, incorporating sophisticated approaches that provide deeper insights into the mechanisms and long-term consequences of exposure across biological levels.

### 4.1 Traditional toxicity endpoints

Conventional *in vivo* assays remain fundamental to MP toxicity evaluation, with model organisms such as the zebrafish (*Danio rerio*), water fleas (*Daphnia magna*), and various mollusks and soil invertebrates widely employed (Cho et al., 2025; Gupta et al., 2023; Lee et al., 2025). These studies typically measure endpoints including acute and chronic mortality, growth inhibition, reproductive impairment, and histopathological alterations in tissues like gills, liver, and intestine. However, their major limitation is low sensitivity to subtle, sublethal effects (e.g., metabolic disruption, immune suppression) that may precede population decline (Afsa et al., 2025; Huang et al., 2025). Additionally, traditional assays often use high exposure concentrations (e.g., >1 mg/L for aquatic invertebrates, >10 mg/kg for terrestrial organisms) (Li et al., 2021; Prata et al., 2022; Liu et al., 2022; Huang et al., 2023) that exceed environmentally relevant levels (ng/L to μg/L or hundreds to tens of thousands of particles per cubic meter in freshwater, 0.1–10 μg/kg or tens to tens of thousands of particles per kilogram in soil) (Zhao SY et al., 2025; Eerkes-Medrano et al., 2015; Liu et al., 2018; Chen et al., 2020). This inconsistency is exacerbated by a lack of standardized dose metrics, which hinders cross-study comparison and mechanistic interpretation (e.g., size-dependent translocation, ROS induction linked to surface area; Chuang et al., 2015). For a unified approach to dose metric selection and reporting, refer to Section 5.1.

### 4.2 Omics technologies

High-throughput omics technologies have revolutionized the mechanistic understanding of MP toxicity by identifying molecular initiating events and altered pathways prior to phenotypic changes. However, interpretation of omics data remains challenging due to issues of reproducibility, context dependence, and linking molecular changes to organismal outcomes.

- Proteomics: MP exposure disrupts key protein-level functions—including metabolic regulation, cellular structural integrity, and stress responses—posing significant risks to organisms. Proteomic analyses enable the large-scale identification and quantification of protein expression changes in MP-exposed organisms, offering system-level insights into toxicity mechanisms. Studies using this approach have revealed widespread metabolic disruptions, such as alterations in energy metabolism (e.g., glycolysis, oxidative phosphorylation) and perturbations in cytoskeletal dynamics (e.g., actin, tubulin dysregulation), impacting cell integrity and function (Liu et al., 2020; Murano et al., 2023; Tang et al., 2023; Zheng et al., 2024). Proteomics also identifies stress-response proteins, including heat shock proteins (HSPs) and oxidative stress markers, detailing cellular responses to MP exposure (Tang et al., 2023; Xie et al., 2025). However, key limitations persist, including the high cost of instrumentation, complex data demands requiring advanced bioinformatics expertise, and the ongoing challenge of correlating protein-level changes with higher-level organismal or ecological outcomes. For instance, while proteomics often identifies oxidative stress markers, few studies confirm whether these markers translate to measurable tissue damage or reduced fitness.
- Transcriptomics and Genomics: Transcriptomics is optimal for exploring MP-induced gene expression perturbations (e.g., pathway-level alterations in immune response, detoxification), while genomics excels at addressing questions about genotoxicity and heritable evolutionary effects. Transcriptomic analyses, particularly RNA sequencing (RNA-Seq), provide comprehensive profiles of gene expression changes following MP exposure. Related studies have revealed alterations in genes related to immune response (e.g., cytokine upregulation), apoptosis, oxidative stress (e.g., SOD, CAT), and detoxification pathways (e.g., cytochrome P450 enzymes) (Chiu et al., 2025; Kazmi et al., 2024; Pedersen et al., 2020). Genomic studies further investigate genotoxic effects and heritable changes, offering insights into long-term evolutionary implications (Gladwell et al., 2025; Wade et al., 2024). However, transcriptomic and genomic analyses face several limitations: (1) there is often a poor correlation between transcriptomic changes and actual protein expression; (2) the methodologies are typically time-consuming and require substantial resources; and (3) the results can be highly context-dependent, influenced by factors such as species biology and specific exposure conditions.

### 4.3 *In silico* and modeling approaches

Computational toxicology is increasingly used to predict MP toxicity, prioritize testing, and enhance risk assessment. Different modeling approaches are tailored to address distinct predictive and integrative questions, with limitations tied to data availability and model simplification.

- Adverse Outcome Pathways (AOPs): Best suited to answer questions about how molecular-level events cascade to organism/population-level adverse outcomes and to identify critical knowledge gaps in toxicity pathways. AOP frameworks organize knowledge into sequences of measurable events, linking Molecular Initiating Events (MIEs, e.g., particle uptake) to Adverse Outcomes (AOs) at organism or population levels (Li YH et al., 2025; Russo et al., 2023). For MPs, AOPs are being developed for outcomes like inflammation and growth impairment, helping identify knowledge gaps and key events for testing (Lan et al., 2025; Rehman et al., 2024). However, fully validated MP-specific AOPs are scarce, and integrating species-specific variability into AOPs remains a major challenge. Additionally, AOPs rely heavily on existing empirical data, which are often inconsistent, limiting their predictive accuracy.
- Toxicokinetic (TK) and Toxicodynamic (TD) Modeling: Ideal for addressing questions about MP absorption, distribution, metabolism, and excretion (ADME) across species, and for extrapolating lab-derived effects to real-world exposure scenarios (Gao N et al., 2024; Qian HL et al., 2025; Wu et al., 2023). These models simulate internal concentrations of MPs and associated chemicals over time, crucial for understanding bioavailability and extrapolating effects across species and exposure scenarios (Chen et al., 2022; Yang et al., 2019). Current model development for MP risk assessment faces key limitations, including an over-reliance on simplifying assumptions about MP behavior, a scarcity of field-validated parameters (e.g., for aged MPs), and inherent challenges in capturing complex environmental interactions, such as MP-pollutant-biological synergy. Dose-response modeling and Quantitative Structure-Activity Relationships (QSAR) models are promising to predict toxicity but also limited by the quality and consistency of input data (e.g., MP property characterization). (Qian HL et al., 2025; Schultz et al., 2021).

### 4.4 Advanced *in vitro* models

Physiologically relevant *in vitro* systems are increasingly used to improve human relevance and mechanistic depth of MP toxicity screening. These include three-dimensional (3D) organoids and organs-on-a-chips (OoCs), which are microfluidic devices that culture cells in perfused chambers to simulate organ-level activities and physiological responses (Abdessalam et al., 2025; Carlsten, 2024; Zhou et al., 2024; Zhou et al., 2023). These models address the core question of how MPs induce organ-specific toxic effects, such as gut barrier dysfunction and hepatotoxicity, within human-relevant physiological microenvironments. For instance, gut-on-a-chip models study epithelial barrier dysfunction and inflammatory responses post-MP ingestion (Ren, 2024), while liver-on-a-chip models assess hepatotoxicity and metabolic disruption caused by MPs and their leachates (Wang YS et al., 2025). These advanced models allow real-time visualization of toxic effects in microenvironments that better mimic human physiology than conventional cell cultures (Wang YS et al., 2025). However, these models are costly and technically complex to fabricate/operate, limiting high-throughput screening. More critically, they cannot fully replicate whole-organism interactions (e.g., systemic immune responses, endocrine feedback loops), meaning results may over- or underestimate. For instance, liver-on-a-chip models may not capture the role of gut-liver crosstalk in MP-induced hepatotoxicity, a key pathway in whole organisms.

## 5 Gaps, actionable regulatory strategies, and future research priorities

Despite significant progress, critical knowledge gaps hinder comprehensive MP risk assessment and effective regulation. Below, we identify these gaps and propose targeted future research priorities, building on the critical evaluation of mechanisms and methods above. This section emphasizes integrative, cross-disciplinary approaches needed to address the limitations of current research.

### 5.1 Methodological standardization

A major obstacle in microplastic (MP) toxicity research is the lack of standardized protocols, which severely hinders regulatory application and reliable risk assessment. This inconsistency spans critical areas: variable MP characterization (e.g., size, shape, polymer verification), divergent experimental designs (exposure concentration, duration), inadequate simulation of environmental aging (e.g., UV irradiation), and unstandardized dose metric selection and reporting. Notably, inconsistent dose metric usage—whether particle number, mass, or surface area—directly obstructs the interpretation of fundamental mechanisms (e.g., particle size effects, translocation, reactive oxygen species (ROS) induction) and undermines cross-study comparability (Rosales and Medina, 2025; Zhang Y et al., 2024). Below, we propose a unified framework and implementation guidelines to address these gaps.

#### 5.1.1 Standardized dose metric framework

Dose metrics (particle number, mass, surface area) must be tailored to research objectives to ensure mechanistic clarity and cross-study comparability:

1. Particle number-based doses (particles/mL or/g): Mandatory for studies investigating physical or biological mechanisms (e.g., membrane translocation, tissue abrasion, oxidative stress induction), as particle number directly reflects surface-area-dependent interactions and bioavailability of MP particles—key drivers of these toxicological effects.
2. Mass-based doses (μg/L or mg/kg): Required for assessing chemical effects (e.g., additive leaching, pollutant adsorption), as mass correlates with total contaminant load (additives + adsorbed pollutants) and cumulative exposure over time—two critical parameters for quantifying chemical toxicity.
3. Surface area-based doses (m^2^/g): Supplementary for mechanistic studies linking particle geometry to toxicity (e.g., ROS generation, cellular uptake efficiency).

This framework resolves longstanding inconsistencies in dose reporting (e.g., conflicting results from mass-vs. number-based dosing in translocation studies) and aligns experimental design with real-world exposure scenarios.

#### 5.1.2 Implementation guidelines

To operationalize this framework and ensure ecological relevance.

1. MP characterization must include polymer type (via FTIR/Raman), size distribution, shape, and aging state to contextualize dose metrics and enable cross-study comparison.
2. Standardized laboratory protocols for simulating environmental weathering (e.g., defined UV irradiation duration) must be integrated to ensure ecological relevance (Yu et al., 2023; Zhang Y et al., 2024).
3. Global cross-laboratory validation studies using reference MP materials are needed to establish inter-lab reproducibility and refine standardized protocols.
4. All studies must report at least one primary dose metric (per research objective) and provide conversions to secondary metrics (e.g., mass-to-particle-number ratios) to facilitate meta-analyses.

### 5.2 Long-term and low-dose exposure studies

Most existing MP toxicity data come from short-term (hours to weeks), high-dose experiments (e.g., 10–1,000 μg/mL *in vitro*, 1–100 mg/L or 0.01–1 mg/d *in vivo*) (Choi et al., 2020; Zheng et al., 2025; Li et al., 2021; Wang et al., 2022), which do not reflect real-world chronic (months to years), low-level exposure (e.g., 203-312 items daily via ingestion of food, water, and dust and inhalation of air for humans) (Cox et al., 2019). To bridge this gap, we recommend: (1) Using environmentally calibrated dose metrics: convert field-measured particle concentrations to particle number per organism per day based on species-specific ingestion rates; (2) Prioritizing mass-based doses for long-term chemical toxicity studies (to quantify cumulative additive/pollutant exposure) and particle number-based doses for physical/biological effects (to capture chronic tissue interaction); and (3) Integrating these metrics with TK models to extrapolate from lab doses to real-world internal exposure. Further studies must focus on chronic MP toxicity, investigating subtle physiological effects (e.g., immunotoxicity, endocrine disruption) that manifest over extended periods (Chen et al., 2025; Marycleopha et al., 2025), with doses anchored to quantitative environmental monitoring data. Transgenerational effects—assessing whether parental MP exposure induces adverse outcomes in unexposed offspring via epigenetic modifications (Chen HB et al., 2024; Talaie et al., 2025)—are also crucial for understanding long-term impacts on population dynamics and ecosystem health. Integrating omics technologies with traditional endpoints in long-term studies will help link molecular changes to persistent organismal effects, addressing the current disconnect between molecular and phenotypic data.

### 5.3 Human health risk assessment

Bridging ecological toxicity data with human health risk assessment remains a key challenge, particularly due to gaps in human-specific exposure and effect pathways. While major human exposure routes (ingestion via seafood/drinking water, inhalation, dermal contact) are identified, the quantification of exposure magnitudes and internal doses across diverse populations (e.g., vulnerable groups such as children or the elderly) is incomplete (Kannan and Vimalkumar, 2021; Rosales and Medina, 2025). To address this, it is crucial to develop robust human exposure models. These models should integrate site-specific data on MP occurrence across the food chain (e.g., seafood trophic levels) and environmental matrices (e.g., drinking water sources, indoor air) with species-specific human TK parameters. Key TK parameters include absorption rates across epithelial barriers, tissue distribution patterns, metabolic transformation, and excretion kinetics (Chen CY et al., 2024; Talaie et al., 2025).

Bridging ecological and human toxicology also requires human-specific regulatory indicators. Mandatory monitoring indicators for human exposure should include:

1. Food: MP concentrations in high-risk items (seafood, bottled water, processed foods) measured via standardized extraction methods like Micro-FTIR;
2. Environmental matrices: Indoor air MP levels and drinking water MP counts) using Raman microspectroscopy.

Two understudied areas demand focused investigation are: (1) the potential for MPs to act as vectors for pathogenic microbes (e.g., antibiotic-resistant bacteria) by facilitating their adhesion and transport into the human body, under specific environmental and epidemiological contexts; and (2) the direct and indirect interactions between ingested MPs and the human gut microbiome—specifically, how MP-induced shifts in microbiome composition (dysbiosis) may disrupt immune function, nutrient metabolism, or increase susceptibility to gastrointestinal diseases (Nissen et al., 2024; Zhi et al., 2024). Translational studies using human-relevant *in vitro* models (e.g., gut-on-a-chip) paired with epidemiological data will improve the relevance of risk assessments to human health.

### 5.4 Advanced technologies

Overcoming current limitations in microplastic (MP) research and effectively translating findings into regulatory action requires the strategic integration of cutting-edge technologies. Machine learning and artificial intelligence can manage complex omics and environmental datasets, identify patterns, and develop predictive toxicity models based on MP properties (size, shape, polymer type) and environmental conditions (Han et al., 2025). These models can reduce reliance on animal testing and directly support regulatory decision-making. Advanced analytical and imaging techniques (e.g., high-resolution confocal microscopy, Raman microspectroscopy) enable *in situ* tracking and identification of MPs in biological tissues and environmental samples, improving understanding of biodistribution and interactions (Le et al., 2025; Ragusa et al., 2022; Wolff et al., 2019). Portable Raman spectrometers and fluorescence-based methods allow for on-site quantification of MPs in water and soil, facilitating real-time environmental compliance checks. Continued integration of multi-omics approaches (transcriptomics + proteomics + metabolomics) will provide a systems-level understanding of MP toxicity mechanisms (Afsa et al., 2025; Huang et al., 2025). Cross-disciplinary collaboration between toxicologists, environmental scientists, data scientists, and clinicians is essential to maximize the utility of these technologies.

## 6 Conclusion

This review underscores the multifaceted threats of microplastics to ecosystems and human health through interconnected physical, chemical, and biological mechanisms. MP toxicity arises from physical properties causing tissue damage, chemical effects via additive leaching and pollutant adsorption leading to synergistic toxicity, and biological disruptions including oxidative stress, genotoxicity, and metabolic alterations. Methodological advances (e.g., omics, computational modeling, advanced imaging techniques) now enable traditional of mechanistic data into actionable regulatory strategies. Future efforts must prioritize methodological standardization, long-term and transgenerational exposure studies, and the integration of ecological data with human health risk assessment. Employing advanced technologies like machine learning and high-resolution imaging will be crucial. A holistic approach combining advanced technologies, standardized methods, and cross-sectoral collaboration is essential to mitigate the global impact of microplastic pollution through evidence-based policies.
